# Wheat Starch Modified with *Ligustrum robustum* (Rxob.) Blume Extract and Its Action Mechanism

**DOI:** 10.3390/foods11203187

**Published:** 2022-10-13

**Authors:** Nan Chen, Hao-Xiang Gao, Qiang He, Wei-Cai Zeng

**Affiliations:** 1 Antioxidant Polyphenols Team, Department of Food Engineering, Sichuan University, Chengdu 610065, China; 2 The Key Laboratory of Food Science and Technology of Sichuan Province of Education, Sichuan University, Chengdu 610065, China

**Keywords:** polyphenol, starch, physicochemical properties, interaction, action mechanism

## Abstract

We investigated the modification of wheat starch with *Ligustrum robustum* (Rxob.) Blume extract (LRE) and determined the action mechanism. Based on differential scanning calorimetry, LRE decreased the gelatinization enthalpy of wheat starch from 19.14 to 7.15 J/g and changed gelatinization temperatures (including the variation in gelatinization onset temperature, peak temperature and conclusion temperature in different degrees). Moreover, LRE affected the pasting viscosity curve of wheat starch, and changed its rheological parameters (including the decrease in storage modulus and loss modulus, as well as the increase in loss tangent). Based on the analysis of scanning electron microscopy and wide-angle X-ray diffraction, LRE increased the hole size and the roughness of the gel microstructure, and decreased the crystallinity of wheat starch. Meanwhile, the evaluation results of the texture analyzer and the colorimeter showed that LRE could change the quality properties (including decrease hardness, fracturability and L* values, as well as increase a* and b* values) of wheat starch biscuits after hot air baking (170 °C). Furthermore, with molecular dynamics simulation analysis, phenolic compounds of LRE combined with starch molecules via H-bonds and affected the formation of molecular bonds (including intra- and intermolecular hydrogen bonds), so as to change the spatial conformation and properties of wheat starch during gelatinization and retrogradation. The present results suggest that LRE can modify the physicochemical properties of wheat starch and further improve its processing properties, indicating its potential in the design and development of starch foods (such as steamed buns, bread, biscuits, etc.).

## 1. Introduction

Starch widely exists in dietary and medicinal plants, and is commonly utilized as a raw material for food products, edible preservative films, drug sustained-release carriers, and so on [1,2]. In order to expand the application of starch, some methods of physical, chemical, and enzymatic treatment are used to modify the properties of starch, so as to improve the natural characteristics of starch and enhance its application value [3]. The chemical modification method by using synthetic reagents is currently most commonly used for preparing modified starch, with the advantages of high efficiency and simple operation [4]. However, the use of synthetic reagents in foods is of concern due to their potential toxicity to human health [3,4]. Therefore, the screening and use of natural compounds for starch modification are important for the development of modified starch and its utilization in the food industry. Phenolic compounds are the secondary metabolites produced by plants, which not only have the advantages of natural and wide sources, but also show the potential to reduce the risk of various diseases (such as certain cancers, type II diabetes and osteoporosis) owing to their multitudinous biological activities (such as anti-oxidation, anti-inflammation and anti-microbial activities) [5]. Moreover, some studies have pointed out that phenolic compounds can affect some properties of starch and the quality of starch food [6,7,8]. Meanwhile, it has been reported that starch can be used as a material for food packaging and drug embedding to carry and protect phenolic compounds [9]. Thus, many efforts are focused on the novel applications of phenolic compounds for starch processing in the food and medicine industries.

*Ligustrum robustum* (Rxob.) Blume is widely planted in southwest China and is a traditional food in China. It has been reported that *L. robustum* is rich in phenolic compounds and exhibits multiple bioactivities, such as anti-hypertension anti-inflammatory, as well as anti-aging activities [10]. Thus, *L. robustum* has wide application prospects in food and other relevant industries.

As part of a series of studies on the valuable utilization of *L. robustum*, in the present work, we sought to modify the physicochemical properties of wheat starch by using *L. robustum* extract (LRE). Furthermore, the potential application of LRE in wheat starch biscuits was evaluated, and molecular dynamics simulation was used to explore the action mechanism of interactions between wheat starch and LRE.

## 2. Materials and Methods

### 2.1. Materials and Reagents

Wheat flour (low gluten, Zhongliang, Beijing, China; protein, fat and carbohydrate contents are 8.0%, 1.6% and 76.5%, respectively) and *L. robustum* (dry leaves) were purchased from a local supermarket in Chengdu, China, and stored at 4 °C. Sodium carbonate, Folin–Ciocalteu reagent, gallic acid, sodium nitrite and rutin were provided by Aladdin (Shanghai, China). All other reagents used were of analytical grade, and the water was purified by a UPR-II-10T pure water instrument (ULUPURE, Chengdu, China). The reagents used for chromatographic analysis were of chromatographic grade.

### 2.2. Preparation of Wheat Starch and L. robustum Extract

The wheat starch was prepared according to the previous study [11,12]. The yield of wheat starch was 70.85% (70.85 g starch/100 g wheat flour). The ratios of amylose and amylopectin in wheat starch were 27.68 ± 1.36% and 70.05 ± 2.58%, respectively [13]. Amylose was composed of glucose units linked by α-1,4 glycoside bonds with a linear structure, and amylopectin was composed of glucose units linked by α-1,4 and α-1,6 glycoside bonds with a branched structure.

The *L. robustum* extract (LRE) was prepared according to the previous studies [14,15]. The yield of LRE was 12.13% (12.13 g extract/100 g sample powder from dry leaves), and with the determination method in our previous study, the total phenol content of LRE was calculated as 178 ± 1.64 mg gallic acid equivalent/g LRE [16]. Meanwhile, according to the previous studies, the main phenolic compounds of LRE were identified as Ligurobustoside B (LGB), Ligurobustoside N (LGN) and Ligupurpuroside J (LPJ) [17,18]. The detailed information on their identification is presented in Supplementary File S1, and the detailed methods for the preparation of wheat starch and LRE are presented in Supplementary File S2.

### 2.3. Effect of LRE on the Physicochemical Properties of Wheat Starch

According to some previous studies, the effect of LRE on the physicochemical properties of wheat starch was determined, including the thermodynamic properties [19], the pasting characteristics [20], rheological properties and gel microstructure [20] and wide-angle X-ray diffraction [21].

The thermodynamic properties of the samples were determined with a Mettler-Toledo DSC (differential scanning calorimeter, Pyris/Diamond, Mettler Toledo International Trading Co., Ltd., Shanghai, China), where 4 μL of LRE solution (0%, 2.5%, 5% and 10%, *w*/*v*) was mixed with 2 mg of wheat starch in the aluminum crucible. The pasting characteristics of the samples were evaluated with an RVA (Rapid Visco Analyzer, Perten Instruments of Australia Pty Ltd., Warriewood, Australia), where LRE was mixed with 3 g of wheat starch to reach the final concentrations of 0%, 5%, 10% and 20% (*w*/*w*, based on the weight of WS). The rheological properties of the samples were determined with an AR G2 stress-controlled rheometer (TA Instruments, New Castle, DE, USA) equipped with a parallel-plate geometry (40 mm in diameter, 1 mm in gap); the samples were taken from the RVA test. The gel microstructure of the samples was observed with a SU8010 SEM (scanning electron microscope, Hitachi, Ltd., Tokyo, Japan) at an accelerating voltage of 15 kV with a magnification of 250 times or 500 times; LRE was added to wheat starch slurry (12%, *w*/*v*) to reach the final concentrations of 5%, 10% and 20% (*w*/*w*, based on the weight of starch in solution). The wide-angle X-ray diffraction of the samples was performed with a D8 Advance wide-angle X-ray diffractometer (Bruker, Ltd., Rheinstetten, German); the sample preparation method was consistent with that in the SEM test. The detailed methods for the determination of the effect of LRE on the physicochemical properties of wheat starch are presented in Supplementary File S2.

### 2.4. Determination of the Quality Properties of Wheat Starch Biscuits with LRE

Briefly, LRE solution (25 mL, 0–5%, *w*/*w*, based on the weight of wheat starch in the mixture) was slowly poured into the wheat starch (50 g) to make a starch block. Subsequently, every 6 g of the starch block was shaped with a mold (4.7 cm × 4.7 cm ×1.7 cm). After that, the shaped starch block was baked for 12 min with hot air (170 °C) and then cooled to 25 °C to produce wheat starch biscuits. The quality properties (including color and texture) of the wheat starch biscuits were determined [22]. The color parameters (L*, a* and b* values) of the biscuits were measured with a colorimeter (Konica Minolta, Chroma Meter, CR400, Tokyo, Japan) standardized by standard white plates. The texture properties (hardness and fracturability values) of the biscuits were determined by a TA-XT2 express texture analyzer (Stable Micro Systems Ltd., Haslemere, U.K.) with a P/2N probe according to the previous study [22]. The detailed methods for the determination of the quality properties of wheat starch biscuits with LRE are presented in Supplementary File S2.

### 2.5. Molecular Dynamics Simulation

Molecular dynamics (MD) simulation is a computational method, which we used to analyze the interaction between LRE and wheat starch [20,23]. Briefly, the starch model (two parallel SGS (short-chain glucose) chains; each SGS chain has three left-handed helixes, which contain a total of 18 D-glucopyranose connected by α-1,4-glycosidic bonds) was built using GLYCAM (https://www.glycam.org, Complex Carbohydrate Research Center, University of Georgia, Athens, GA, USA). Phenolic components identified from LRE (including LGB, LGN and LPJ) and reaction solvent (water box: TIP3PBOX, size: 65 × 46 × 49 Å3) were built and optimized for MD simulation. Thereafter, the starch model with the GLYCAM-06j-1 force field and LGB (LGN or LPJ) with the generalized amber force field (GAFF) were loaded into the water solvent box in AMBER software. Then, the energy minimization procedure was used to reduce system energy. The NVT (canonical ensemble) procedure was carried out to increase (from 0 to 370 K) or decrease (from 370 to 277 K) the system temperature for simulating the gelatinization and retrogradation of starch, respectively. Then, the NPT (constant molecule, pressure and temperature) procedure was used to balance the simulated system. Finally, the production run procedure was used to simulate the interactions between SGS and LGB (LGN or LPJ) at a simulated gelatinization temperature (370 K) or simulated retrogradation temperature (277 K). The trajectory of MD simulation, intramolecular H-bond, intermolecular H-bond, the center of mass (COM) distance and root mean square deviation (RMSD) were separately observed, recorded and calculated. The detailed simulation method is presented in Supplementary File S2.

### 2.6. Statistical Analysis

The data from triplicate analyses are expressed as mean ± standard deviation (SD). SPSS 22.0 software (SPSS Inc., Chicago, IL, USA) was employed for analysis of variance (ANOVA), and the significant differences (*p* < 0.05) were determined by using Tukey’s test.

## 3. Results and Discussion

### 3.1. Thermodynamic Properties of Wheat Starch with LRE

As presented in Table 1, *T_o_* (gelatinization onset temperature), *T_p_* (peak temperature), *T_c_* (conclusion temperature) and Δ*Hg* (gelatinization enthalpy) of wheat starch were changed by LRE. Notably, the Δ*Hg* value decreased with the increase in LRE concentration, especially the addition of 20% LRE (*w*/*w*, based on the weight of starch). Δ*Hg* represents the energy absorbed by molecules or chain segments to leave the lattice, which is related to the strength of intermolecular force. The greater the intermolecular force, the greater the Δ*Hg* value [24,25]. It has been reported that the gelatinization of starch granules may be attributed to the rupture of the amylopectin double helices and the melting of the crystalline lamellae, which need high temperature and energy to break the strong bonds between starch molecules [24,25]. In the present study, LRE could promote the gelatinization of wheat starch, which might be attributed to the hydroxyl groups in phenolic compounds of LRE. It has been reported that the hydroxyl groups of phenolic compounds may bind to starch chains through hydrogen bonds, which may reduce the tightness of starch crystalline micelles. The crystalline micelle structure is maintained by the molecular bonds (such as hydrogen bonds) among starch molecules. The decrease in the molecular forces among starch molecules indicates that starch granules are more easily destroyed with a low Δ*Hg* value. When 20% LRE was co-gelatinized with starch, LRE not only promoted the movement of water molecules into starch granules, but also interacted with starch by a large number of multi-point hydrogen bonds, which could weaken the molecular bonds among starch molecules and decrease the Δ*Hg* value [26].

### 3.2. Pasting Characteristics of Wheat Starch with LRE

**Figure 1:**
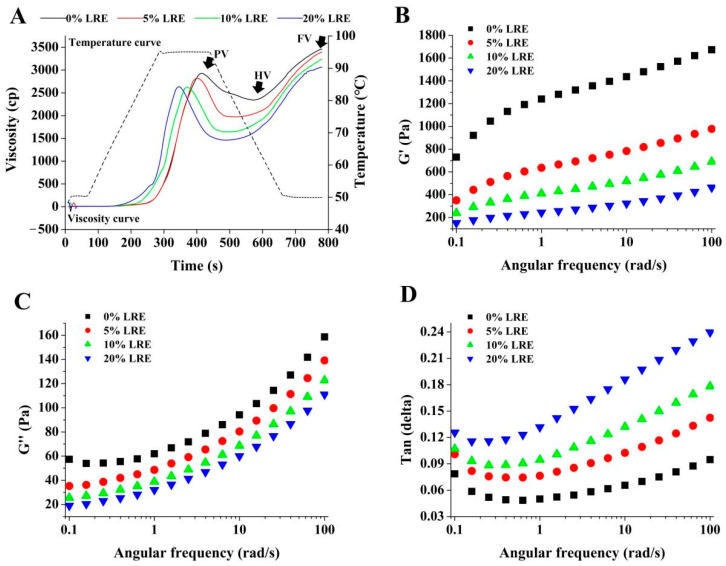
Effect of LRE on the pasting and rheological properties of wheat starch. (**A**) Pasting property, (**B**) storage modulus (G′), (**C**) loss modulus (G″), (**D**) loss tangent (Tanδ). LRE: *Ligustrum robustum* (Rxob.) Blume extract.

As presented in Figure 1A, LRE changed the pasting viscosity curve of wheat starch and affected its pasting process. The values of key parameters of the viscosity curve are recorded in Table 1. As shown in Table 1, LRE caused a decline in peak viscosity (PV), hold viscosity (HV), final viscosity (FV) and pasting time (PT) of wheat starch, whereas breakdown (BD) increased, and all were concentration-dependent. Relevant research reported that phenolic compounds can promote the swelling of starch granules in the pasting process, thus decreasing the PV value of starch paste [6,27]. Meanwhile, phenolic compounds can affect the winding and alignment of starch molecule chains, which can lead to the variation in HV and FV of samples [21]. Moreover, an increase in BD value indicates a decline in granule integrity, while a lower PT value indicates a decrease in pasting time and an advancement in pasting progress.

### 3.3. Rheological Properties of Wheat Starch with LRE

The G′ (storage modulus, Figure 1B), G″ (the loss modulus, Figure 1C) and Tanδ (loss tangent, Figure 1D) of samples are recorded in Figure 1. Notably, the value of G″ was far lower than that of G′ for all testing samples, and this phenomenon indicated that the elasticity of all samples was dominant compared to the viscosity of samples. Moreover, G′ and G″ curves for all testing samples showed an upward trend as angular frequency increased, and this phenomenon showed that both the elasticity and viscosity of samples were frequency-dependent. In addition, with the increase in LRE concentration, G′ and G″ both decreased, which indicated that LRE could affect the formation of the starch gel network. Meanwhile, the result of Tanδ (Tanδ < 1, Figure 1D) suggested that the deformation of samples was basically recoverable, and it behaved more like a solid. Moreover, the Tanδ value increased due to the addition of LRE in the set angular frequency range; this phenomenon showed the weakening of solid-like behavior [28].

### 3.4. Gel Microstructure of Wheat Starch with LRE

**Figure 2:**
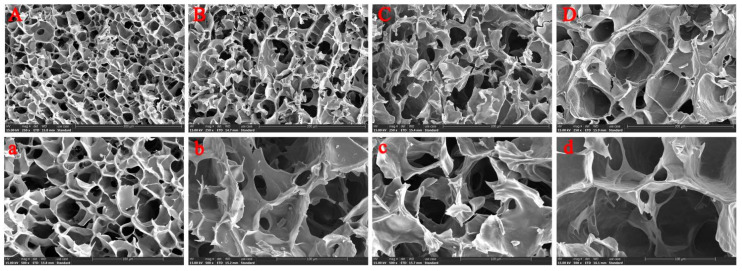
SEM images of wheat starch gel with different concentrations of LRE. (**A**,**a**) Control group. (**B**,**b**) 5% LRE group. (**C**,**c**) 10% LRE group. (**D**,**d**) 20% LRE group. (Capital letters represent the magnification of 250 times; lowercase letters represent the magnification of 500 times.) LRE: *Ligustrum robustum* (Rxob.) Blume extract.

As shown in Figure 2, the control starch gel (without LRE) formed a dense network structure, and the interlayers were cross-linked to form many relatively uniform pores (Figure 2A,a). With the addition of LRE, the hole size of the network structure in the starch gel increased and the cross-linking degree among starch gel layers decreased, which were concentration-dependent (Figure 2B,b–D,d). In addition, compared with the control (Figure 2a), the roughness of the gel surface increased (Figure 2b–d), which might be attributed to the embedding of LRE in the starch molecules.

### 3.5. Crystalline Form and Recrystallization Degree of Wheat Starch with LRE

**Figure 3:**
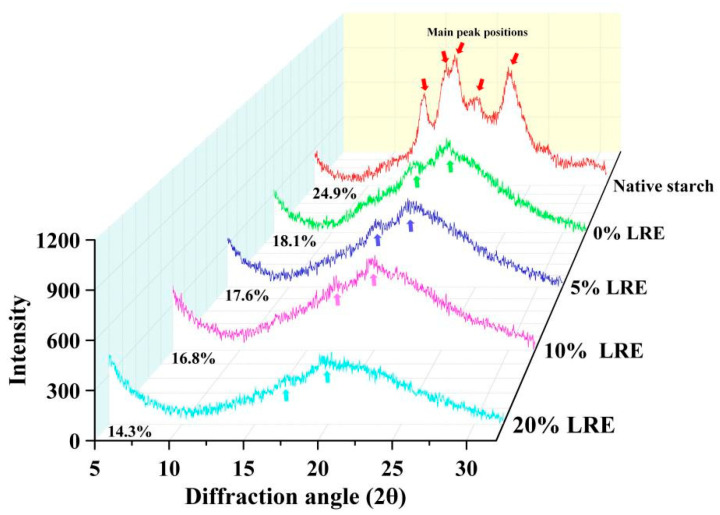
Wide-angle X-ray diffraction pattern of wheat starch gel with different concentrations of LRE. LRE: *Ligustrum robustum* (Rxob.) Blume extract.

As presented in Figure 3, an A-type crystal structure with strong peaks at 2θ~15°, 17°, 18° and 23° was exhibited in native wheat starch, and this peak type was generally regarded as the typical WAXD (wide-angle X-ray diffraction) pattern of cereal starch [21]. After full gelatinization and short-term regeneration, the original WAXD pattern was changed from an A-type diffraction pattern to a mixed diffraction pattern of B-type and V-type, and this phenomenon might be due to the destruction of the semi-crystalline structure in native starch after gelatinization and re-formation of the ordered crystal structure after short-term retrogradation [29]. Meanwhile, B-type pattern and V-type pattern of wheat starch presented well-defined peaks around 2θ~17° and 20°, respectively. The addition of LRE did not change the position of the diffraction peak, but the interaction between LRE and starch decreased the intensity of diffraction peaks of starch, which was concentration-dependent. Recent research points out that phenolic compounds can hinder recrystallisation among starch molecules, which can weaken the B-type and V-type crystal structure [18]. Moreover, the native wheat starch showed a semi-crystalline structure with higher relative crystallinity (24.9%), while the wheat starch with LRE showed a lower relative crystallinity (17.6%, 16.8% and 14.3% for the 5%, 10% and 20% LRE groups, respectively) than that of wheat starch without LRE (18.1%). The previous study also reported that phenolic compounds can destroy the hydrophobic interactions among starch chains via H-bonds, thus reducing the chance of starch molecules forming a double-helical structure [30].

### 3.6. Quality Properties of Wheat Starch Biscuit with LRE

As presented in Table 2, the addition of 1% LRE increased the L* value and decreased the a* value of samples, whereas the addition of a higher concentration (2% to 5%) of LRE decreased the L* value and sightly increased the a* value of samples. Moreover, the addition of LRE significantly increased the b* value of samples. As reported in the previous study, *L. robustum* is rich in phenolic compounds, and the oxidative polymerization of phenolic compounds can produce pigments, which may contribute to the color changes [31,32]. Furthermore, as presented in Table 2, LRE could decrease the value of hardness (from 1829.63 to 873.89) and fracturability (from 11,053.71 to 7703.13) of all wheat starch biscuit samples, being concentration-dependent. Commonly, gelatinization and retrogradation have critical roles in the quality of starch. The starch gel will be formed during the gelatinization and retrogradation processes. The rheological properties of starch gel are important for the taste and quality of starch food. The network microstructure of starch gel is related to the above gel properties. The present results indicate that LRE could change the thermal properties, pasting characteristics, rheological properties, microstructure and crystallinity of wheat starch after gelatinization and short-term retrogradation, so as to potentially affect the texture (including the hardness and fracturability) of wheat starch biscuits.

### 3.7. Molecular Interaction between Starch and LRE

**Figure 4:**
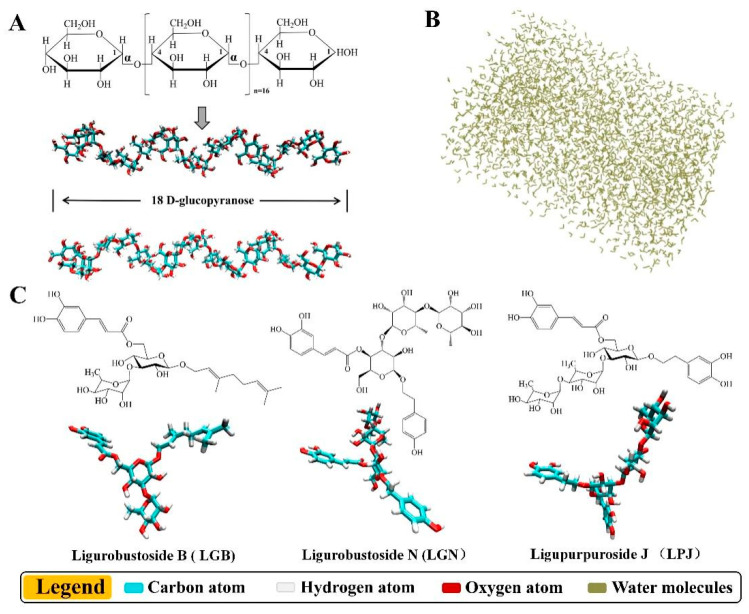
Molecular structure models for molecular dynamics simulation. (**A**) Two parallel SGS (short-chain glucose) chains, (**B**) TIP3PBOX water solvent box, (**C**) phenolic compounds identified from LRE: Ligurobustoside B (LGB), Ligurobustoside N (LGN) and Ligupurpuroside J (LPJ). LRE: *Ligustrum robustum* (Rxob.) Blume extract.

**Figure 5:**
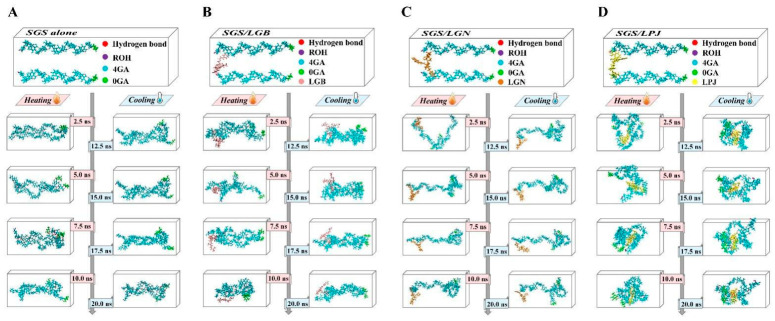
Trajectory of molecular dynamics simulation. (**A**) Trajectory between two SGS chains, (**B**) trajectory between LGB and two SGS chains, (**C**) trajectory between LGN and two SGS chains, (**D**) trajectory between LPJ and two SGS chains. (The glucose residue at the beginning of starch stand is 0GA, the glucose residue in the middle of starch strand is 4GA and the hydroxyl group at the end of starch strand is ROH.) LRE: *Ligustrum robustum* (Rxob.) Blume extract.

As shown in Figure 4, the starch model (two parallel SGS (short-chain glucose) chains, Figure 4A), the TIP3PBOX model (Figure 4B), as well as phenolic compounds of LRE (LGB, Ligurobustoside B; LGN, Ligurobustoside N; and LPJ, Ligupurpuroside J; Figure 4C) were successfully established. As shown in Figure 5A, two parallel SGS chains were initially separated from each other. During the heating process (0–10 ns), the winding and binding of two SGS chains were caused by the formation and dissociation of H-bonds (Table 3) in two SGS chains. Meanwhile, owing to the variation in hydrogen bonding sites (Table 3), the winding conformation of two SGS chains was in a dynamic change. Then, as system temperature and energy decreased, the fluctuation of binding conformation in two SGS chains was relatively small in the cooling process (10–20 ns) and maintained a helical structure via the stable H-bond network (Table 3). The trajectory between the starch model and phenolic compounds of LRE (LGB, LGN and LPJ) is shown in Figure 5B–D. During heating time and cooling time, the winding conformation between two SGS chains was significantly changed due to the presence of phenolic compounds of LRE. As shown in Figure 5B, two SGS chains twined around each other to form a tight binding structure, whereas LGB only combined with the outer side of the associated structure formed by two SGS chains. Moreover, it can be seen from Figure 5C that LGN hindered the combination of two SGS chains by connecting with the reducing end of a single SGS chain, and LGN occupied the H-bond binding site (Table 4) of two SGS chains, thereby inhibiting the combination of two SGS chains. However, as shown in Figure 5D, the combination of LPJ and SGS double chains was tighter than that in the SGS/LGB group and the SGS/LGN group. The LPJ was completely embedded in the cavity formed through two SGS chains, and this combination was kept via continuous intermolecular H-bonds (Table 4). Notably, the complex effects of molecule size, steric hindrance and phenolic hydroxyl amounts of LGB, LGN and LPJ lead to their variations in binding conformation and binding capability. As shown in Figure 4C, the relative molecular masses of LGB (molecular formula: C31H44O13), LGN (molecular formula: C35H46O18) and LPJ (molecular formula: C35H46O19) were 624, 754 and 770, respectively. The molecule size and steric hindrance of LGB were smaller than those of LGN (or LPJ), and the hydroxyl group amount of LGB was also the lowest. In addition, the molecular conformation of LGN and LPJ was similar, but the amount of pyrocatechol groups of LPJ was more than that of LGN, which might cause the difference in their binding affinity with starch molecules.

**Figure 6:**
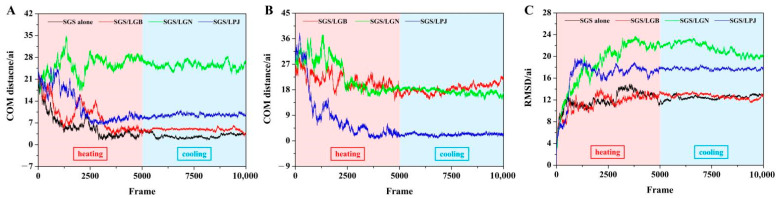
(**A**) COM distance between two SGS chains, (**B**) COM distance between two SGS chains and phenolic compounds of LRE, (**C**) RMSD value of different interaction system. (Total simulation time: 2 × 10^−8^ s, total frames: 10,000. “ai”: the unit of length. 1 ai = 1 × 10^−10^ m. Heating process: 0 to 4999 frames, temperature was set at 370 K. Cooling process: 5000 to 10,000 frames, temperature was set at 277 K.) LRE: *Ligustrum robustum* (Rxob.) Blume extract.

For evaluating the status of the above simulation trajectory, the center of mass (COM) distance and root mean square deviation (RMSD) value were calculated and recorded. COM distance reflects the dynamic distance between molecules and the binding state, and RMSD value reflects the rationality and stability of the present molecular dynamics simulation. As shown in Figure 6A, the COM distance value between two SGS chains gradually decreased and finally tended to be stable in the control group, and the presence of phenolic compounds of LRE (LGB, LGN and LPJ) increased the COM distance value between two SGS chains, but the effect of LGN was more obvious, which might be due to the larger steric hindrance and lower hydroxyl group amount of LGN. Then, the COM distance between two SGS chains and phenolic compounds of LRE was observed and is shown in Figure 6B. The COM distance value between LPJ and two SGS chains was much lower than that of other groups, which indicated that the combination of LPJ and two SGS chains was tighter. Meanwhile, the RMSD value was recorded. Figure 6C shows that the RMSD value of four simulation systems firstly increased, and then tended to be stable at last. Commonly, the equilibrium of the RMSD value indicates that the dynamic combination among molecules reaches a relative stability and that the design of simulation systems is feasible [33].

Furthermore, the position and amount of H-bonds (including intra- and intermolecular H-bonds) in the present MD simulation were calculated. As presented in Table 3, phenolic compounds of LRE (LGB, LGN and LPJ) altered the position and amount of intramolecular H-bonds of two SGS chains both in heating and cooling processes. Usually, the intramolecular hydrogen bonds with high occupancy are especially key to keep the SGS-wound form. The highest ratios (33.0%, 1650 frames in 5000 frames of heating time; 91.9%, 4593 frames in 5000 frames of cooling time) of intramolecular H-bonds were both observed in the LPJ/SGS group, which indicated that LPJ significantly affected the formation of intramolecular H-bonds within different periods. In addition, the distributions of intermolecular H-bonds between two SGS chains and phenolic compounds of LRE (LGB, LGN and LPJ) were further recorded. As shown in Table 4, between the hydroxyl groups in phenolic compounds of LRE and the glycan hydroxyl groups of SGS, intermolecular H-bonds could form. During the heating time, the intermolecular hydrogen bonds’ occupancy in LGB/SGS, LGN/SGS and LPJ/SGS groups was no more than 10% due to the high energy and high temperature. However, the average occupancy of intermolecular hydrogen bonds in the LPJ/SGS group was higher than that of others, and the intermolecular hydrogen bonds were more continuous. During cooling time, the combination between two SGS chains and LPJ was more stable by the continuous intermolecular hydrogen bonds and the high hydrogen bond occupancy ratio (from 37.2% to 88.0%).

## 4. Conclusions

In the present study, we investigated the modification of wheat starch with LRE and explored the action mechanism. According to the determination results, some physicochemical properties of wheat starch, such as thermodynamics properties, pasting characteristics, rheological properties, gel microstructure and crystallinity, were modified by LRE. Meanwhile, LRE also showed the potential to change the quality properties (including color and texture) of wheat starch biscuits. Then, molecular dynamics (MD) simulation was employed to analyze the action mechanism and provide some useful references for the effect of LRE modification on the physicochemical properties of wheat starch. MD simulation indicated that phenolic compounds of LRE interacted with starch molecules, destroyed the intramolecular H-bonds between starch molecules and formed intermolecular H-bonds with starch molecules, thereby changing the spatial configuration of starch molecule chains and affecting the properties of starch during gelatinization and retrogradation. Moreover, the complex impact of molecule size, steric hindrance and phenolic hydroxyl amount of LGB, LGN and LPJ led to their variations in binding conformation and binding capability with the starch model, and of the three phenolic compounds, LPJ exhibited the most significant capability to interact with the starch model. The results suggest that LRE has the potential to improve the properties of wheat starch in the food and chemical industries. Further studies (including the nutritional characteristics and bioactivities of starch products) are underway to elucidate the valuable applications of wheat starch modified by LRE in food and chemical industries.

## Figures and Tables

**Table 1:** The effect of LRE on the thermodynamic properties and pasting characteristics of wheat starch.

Concentration of LRE	Gelatinization Parameter				Pasting Parameter				
	*T_o_* (°C)	*T_p_* (°C)	*T_c_* (°C)	Δ*Hg* (J/g)	PV (cp)	HV (cp)	FV (cp)	BD (cp)	PT (min)
0%	57.46 ± 0.56 ^ab^	62.20 ± 0.36 ^ab^	67.09 ± 0.24 ^a^	19.14 ± 0.32 ^a^	2927 ± 2 ^a^	2221 ± 3 ^a^	3459 ± 3 ^a^	706 ± 4 ^d^	6.93 ± 0.3 ^a^
5%	56.43 ± 0.13 ^b^	61.70 ± 0.21 ^bc^	66.34 ± 0.22 ^c^	17.13 ± 0.41 ^b^	2821 ± 3 ^b^	1971 ± 2 ^b^	3442 ± 2 ^b^	850 ± 1 ^c^	6.47 ± 0.1 ^b^
10%	56.12 ± 0.22 ^bc^	61.91 ± 0.11 ^b^	66.57 ± 0.14 ^bc^	15.24 ± 0.37 ^c^	2626 ± 2 ^cd^	1642 ± 3 ^c^	3232 ± 2 ^bc^	984 ± 3 ^b^	6.20 ± 0.2 ^bc^
20%	57.55 ± 0.37 ^a^	62.74 ± 0.32 ^a^	66.78 ± 0.26 ^b^	7.15 ± 0.42 ^d^	2634 ± 3 ^c^	1461 ± 4 ^d^	3061 ± 1 ^c^	1173 ± 3 ^a^	5.73 ± 0.1 ^c^

Each value is expressed as mean ± SD (*n* = 3). Different superscript letters in each vertical column denote statistically significant differences (*p* < 0.05). Δ*Hg* was calculated based on normalization to the starch mass. PV: peak viscosity, HV: hold viscosity, FV: final viscosity, BD: breakdown, PT: pasting time, LRE: *Ligustrum robustum* (Rxob.) Blume extract.

**Table 2:** Color and texture of biscuits baked from wheat starch gel and LRE in different concentrations.

Concentration of LRE	L*	a*	b*	Hardness (g)	Fracturability (g·s)
0%	88.88 ± 2.23 ^ab^	−0.35 ± 0.03 ^cd^	10.40 ± 1.81 ^f^	1830 ± 56 ^a^	11,054 ± 575 ^a^
1%	89.91 ± 1.59 ^a^	−0.58 ± 0.05 ^e^	12.27 ± 1.65 ^e^	1709 ± 129 ^b^	8712 ± 361 ^b^
2%	87.42 ± 2.72 ^b^	−0.48 ± 0.09 ^d^	19.53 ± 1.62 ^d^	1550 ± 106 ^c^	8559 ± 772 ^bc^
3%	85.01 ± 1.46 ^c^	−0.28 ± 0.01 ^c^	24.43 ± 1.80 ^c^	1413 ± 184 ^d^	8402 ± 247 ^cd^
4%	84.60 ± 0.98 ^cd^	−0.06 ± 0.10 ^b^	26.71 ± 1.00 ^b^	1262 ± 87 ^e^	8451 ± 501 ^c^
5%	83.73 ± 0.60 ^d^	0.04 ± 0.13 ^a^	29.83 ± 1.37 ^a^	874 ± 90 ^f^	7703 ± 258 ^d^

Each value is expressed as mean ± SD (*n* = 3). Different superscript letters in each vertical column denote statistically significant differences (*p* < 0.05). LRE: *Ligustrum robustum* (Rxob.) Blume extract.

**Table 3:** The intramolecular hydrogen bonds within two SGS chains in different interaction systems.

Interaction System	Simulation Phase	Hydrogen Bond Acceptor		Hydrogen Bond Donor		Frames	Ratio (%)
		Acceptor Molecule	Acceptor Atom	Donor Molecule	Donor Atom		
SGS alone	Heating	SGS	4GA_25@O2	SGS	4GA_4@H6O	606	12.1
		SGS	4GA_25@O5	SGS	4GA_7@H3O	545	10.9
		SGS	4GA_25@O6	SGS	4GA_7@H3O	364	7.3
		SGS	4GA_30@O5	SGS	4GA_12@H3O	310	6.2
		SGS	4GA_25@O6	SGS	4GA_7@H2O	282	5.7
	Cooling	SGS	4GA_28@O2	SGS	4GA_7@H6O	2560	51.2
		SGS	4GA_27@O2	SGS	4GA_7@H3O	2356	47.1
		SGS	4GA_37@O2	SGS	4GA_15@H2O	2322	46.4
		SGS	4GA_35@O6	SGS	4GA_16@H2O	2048	40.9
		SGS	4GA_30@O5	SGS	4GA_12@H3O	1659	33.2
LGB/SGS group	Heating	SGS	4GA_28@O2	SGS	4GA_27@H3O	1157	23.1
		SGS	4GA_9@O2	SGS	4GA_8@H3O	1063	21.2
		SGS	4GA_8@O2	SGS	4GA_7@H3O	1048	20.9
		SGS	4GA_7@O6	SGS	4GA_29@H2O	960	19.2
		SGS	4GA_5@O2	SGS	4GA_4@H3O	959	19.2
	Cooling	SGS	4GA_7@O6	SGS	4GA_29@H2O	4367	87.3
		SGS	0GA_39@O2	SGS	4GA_19@H2O	3960	79.2
		SGS	4GA_26@O5	SGS	4GA_25@H6O	3902	78.0
		SGS	4GA_24@O5	SGS	4GA_29@H3O	3493	69.9
		SGS	4GA_28@O2	SGS	4GA_27@H3O	3340	66.8
LGN/SGS group	Heating	SGS	4GA_9@O2	SGS	4GA_8@H3O	1298	26.0
		SGS	4GA_19@O2	SGS	4GA_18@H3O	1182	23.6
		SGS	4GA_14@O2	SGS	4GA_13@H3O	943	18.9
		SGS	4GA_4@O5	SGS	4GA_3@H6O	885	17.7
		SGS	4GA_18@O2	SGS	4GA_17@H3O	873	17.5
	Cooling	SGS	4GA_8@O2	SGS	4GA_38@H2O	4076	81.5
		SGS	0GA_39@O2	SGS	4GA_14@H3O	3808	76.2
		SGS	4GA_36@O3	SGS	4GA_15@H3O	3022	60.4
		SGS	4GA_13@O2	SGS	4GA_12@H3O	2981	59.6
		SGS	4GA_8@O2	SGS	4GA_38@H3O	2761	55.2
LPJ/SGS group	Heating	SGS	4GA_29@O2	SGS	4GA_28@H3O	1650	33.0
		SGS	4GA_30@O2	SGS	4GA_29@H3O	1480	29.6
		SGS	4GA_36@O2	SGS	4GA_35@H3O	1340	26.8
		SGS	4GA_4@O2	SGS	4GA_25@H6O	1256	25.1
		SGS	4GA_18@O2	SGS	4GA_17@H3O	1181	23.6
	Cooling	SGS	4GA_38@O2	SGS	4GA_19@H2O	4593	91.9
		SGS	4GA_37@O3	SGS	4GA_12@H3O	4330	86.6
		SGS	4GA_18@O2	SGS	4GA_17@H3O	4259	85.2
		SGS	4GA_17@O2	SGS	4GA_16@H3O	3858	77.2
		SGS	0GA_20@O2	SGS	4GA_19@H3O	3436	68.7

SGS: short-chain glucose; LGB: Ligurobustoside B; LGN: Ligurobustoside N; LPJ: Ligupurpuroside J. The 0GA and 4GA represent the glucose residue at the beginning and in the middle of SGS, respectively. The numbers after 4GA (or 0GA) represent the residue sequence number in molecule. H2O (or H3O, H6O) and O2 (or O3, O5, O6) after the @ symbol are the representations of the hydrogen atom and oxygen atom of SGS in the force field, respectively. The amount and position of hydrogen bonds of the top 5 occupancies in each interaction system are counted in the table; others are not included in the table. LRE: *Ligustrum robustum* (Rxob.) Blume extract.

**Table 4:** The intermolecular hydrogen bonds between two SGS chains and the phenolic compounds of LRE.

Interaction System	Simulation Phase	Hydrogen Bond Acceptor		Hydrogen Bond Donor		Frames	Ratio (%)
		Acceptor Molecule	Acceptor Atom	Donor Molecule	Donor Atom		
LGB/SGS group	Heating	LGB	LGB_1@O1	SGS	4GA_26@H2O	464	9.3
		LGB	LGB_1@O7	SGS	4GA_5@H3O	380	7.6
		SGS	4GA_29@O2	LGB	LGB_1@H5	355	7.1
		SGS	4GA_25@O6	LGB	LGB_1@H4	276	5.5
		SGS	4GA_7@O2	LGB	LGB_1@H5	245	4.9
	Cooling	SGS	4GA_29@O2	LGB	LGB_1@H5	4759	95.1
		SGS	4GA_25@O6	LGB	LGB_1@H4	1702	34.0
		LGB	LGB_1@O13	SGS	4GA_8@H2O	1605	32.1
		LGB	LGB_1@O1	SGS	4GA_26@H2O	866	17.3
		SGS	4GA_7@O3	LGB	LGB_1@H26	658	13.2
LGN/SGS group	Heating	LGN	LGN_1@O14	SGS	4GA_24@H3O	472	9.4
		SGS	4GA_25@O2	LGN	LGN_1@H19	354	7.1
		LGN	LGN_1@O11	SGS	4GA_25@H2O	321	6.4
		LGN	LGN_1@O10	SGS	4GA_24@H3O	262	5.2
		LGN	LGN_1@O14	SGS	4GA_25@H2O	258	5.1
	Cooling	SGS	4GA_23@O3	LGN	LGN_1@H31	220	4.4
		SGS	4GA_24@O3	LGN	LGN_1@H19	146	2.9
		SGS	4GA_25@O2	LGN	LGN_1@H35	119	2.4
		SGS	4GA_23@O2	LGN	LGN_1@O18	59	1.2
LPJ/SGS group	Heating	LPJ	LPJ_1@O3	SGS	4GA_11@H3O	457	9.1
		LPJ	LPJ_1@O6	SGS	4GA_11@H2O	451	9.0
		SGS	4GA_11@O3	LPJ	LPJ_1@H15	451	9.0
		LPJ	LPJ_1@O7	SGS	4GA_4@H2O	438	8.8
		LPJ	LPJ_1@O17	SGS	4GA_5@H6O	406	8.1
	Cooling	SGS	4GA_12@O2	LPJ	LPJ_1@H5	4402	88.0
		LPJ	LPJ_1@O3	SGS	4GA_18@H2O	3253	65.1
		LPJ	LPJ_1@O18	SGS	4GA_18@H3O	2465	49.3
		SGS	4GA_32@O2	LPJ	LPJ_1@H16	2174	43.5
		LPJ	LPJ_1@O17	SGS	4GA_5@H6O	1860	37.2

SGS: short-chain glucose; LGB: Ligurobustoside B; LGN: Ligurobustoside N; LPJ: Ligupurpuroside J. The 4GA represents the glucose residue in the middle of SGS. The numbers after 4GA (or LGB, LGN, LPJ) represent the residue sequence number in the molecule. H2O (or H3O, H6O, H5, H15, H16, H19, et al.) and O1 (or O2, O3, O5, O6, et al.) after the @ symbol are the representations of the hydrogen atom and oxygen atom of SGS (or LGB, LGN, LPJ) in the force field, respectively. The amount and position of hydrogen bonds of the top 5 occupancies in each interaction system are counted in the table; others are not included in the table. LRE: *Ligustrum robustum* (Rxob.) Blume extract.

## Data Availability

All of the data are contained within the article and the Supplementary Materials.

## Supplementary Materials

The following supporting information can be downloaded at: https://www.mdpi.com/article/10.3390/foods11203187/s1, Supplementary Files S1 and S2.
