# Supplementary material for: Wheat Starch Modified with Ligustrum robustum (Rxob.) Blume Extract and Its Action Mechanism
Source: Foods. 2022 Oct 13;11(20):3187. doi: 10.3390/foods11203187 (PMC9601434; doi:10.3390/foods11203187)
Supplement: Supplementary file 1 [file foods-11-03187-s001.zip › Supplementary File S1.pdf]

Supplementary materials

Using high performance liquid chromatography-mass spectrum (HPLC-MS, Agilent 1290-6460 HPLC System, Agilent Technologies, Inc., CA, USA) and nuclear magnetic resonance (NMR, 400-MR DD2 spectrometer, Agilent Technologies Inc., CA, USA), the main active compounds of LRE were identified to be Ligurobustoside B (LGB), Ligurobustoside N (LGN) and Ligupurpuroside J (LPJ), respectively.

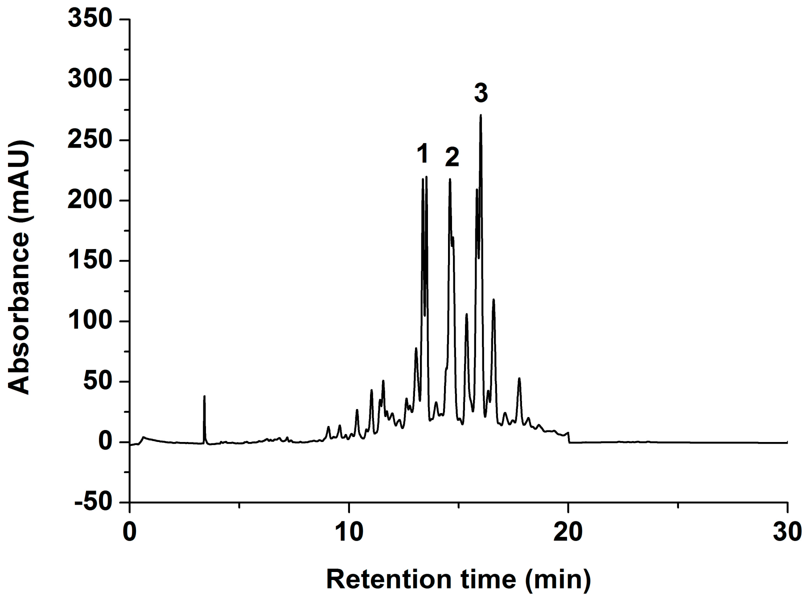

Figure S1. The results of High performance liquid chromatography (HPLC)

Methanol (A) and deionized water with 0.1% formic acid (B) were used as mobile phase. Gradient elution was set as: 0-10 min, 0-50% A; 10-15 min, 50-60% A; 15-20 min, 60-70% A; 20-25 min, 70-80% A; 25-30 min, 80-90% A; 35-40 min, 90-100% A; 45-60 min, 100% A. The analytical conditions were the following: column temperature, 25 °C; flow rate, 1 mL/min; injection volume, 20 μL (the concentration of LRE: 10 μg/μL), and detection, 320 nm. The mass spectra were performed using TSQ Quantum Ultra (Thermo, USA) with an electrospray source operating in negative ion mode and a mass spectrum scanning range from 50 to 1200 m/z.

1. The results of mass spectrum (MS) and nuclear magnetic resonance (NMR)

Compound 1：Ligurobustoside B (LGB)

ESI-MS m/z: 623 $[M-H]^-$, Molecular formula: $C_{31}H_{44}O_{13}$

^{1}H-NMR (CD_3OD, 600 MHz) δ: 7.62 (1H, d, *J*=15.9 Hz, H-7'''), 7.08 (1H, d, *J*=2.3 Hz, H-2'''), 6.94 (1H, dd, *J*=8.3, 2.1 Hz, H-5'''), 6.82 (1H, d, *J*=8.1 Hz, H-6'''), 6.23 (1H, d, *J*=15.7 Hz, H-8'''), 5.38 (1H, t, *J*=7.1 Hz, H-2), 5.06 (1H, t, *J*=6.5 Hz, H-6), 4.39 (1H, dd, *J*=12.1, 7.3 Hz, H-1a), 4.22 (1H, dd, *J*=11.5, 7.3 Hz, H-1b), 2.15 (2H, t, *J*=6.5 Hz, H-5), 1.97 (2H, t, *J*=7.5 Hz, H-4), 1.74 (6H, s, H-8, 10), 1.58 (3H, s, H-9)

^{13}C-NMR (CD_3OD, 150 MHz) δ: 168.6 (C=O), 150.2 (C-4'''), 148.2 (C-8'''), 147.1 (C-3'''), 141.9 (C-3), 133.6 (C-7), 127.4 (C-1'''), 125.7 (C-6), 123.5 (C-6'''), 121.3 (C-2), 116.8 (C-5'''), 115.1 (C-2'''), 114.6 (C-7'''), 103.4 (C-1'), 102.3 (C-1''), 82.1 (C-3'), 76.5 (C-2', 5'), 73.6 (C-4''), 72.3 (C-2''), 71.9 (C-3''), 70.6 (C-4'), 69.9 (C-5''), 67.2 (C-1), 62.8 (C-6'), 41.1 (C-4), 27.3 (C-5), 25.7 (C-8), 18.8 (C-6''), 17.4 (C-9), 16.3 (C-10)

Compound 2：Ligurobustoside N (LGN)

ESI-MS m/z: 753 $[M-H]^-$, Molecular formula: $C_{35}H_{46}O_{18}$

^{1}H-NMR (CD_3OD, 600 MHz) δ: 7.81 (1H, d, *J*=15.7 Hz, H-7''''), 7.32 (2H, d, *J*=8.7 Hz, H_2-2, 6), 7.28 (1H, d, *J*=2.2 Hz, H-2''''), 7.17 (1H, dd, *J*=2.2, 8.3 Hz, H-6''''),7.05 (1H, d, *J*=8.3 Hz, H-5''''), 6.92 (2H, d, *J*=8.7 Hz, H_2-3, 5), 6.52 (1H, d, *J*=15.7 Hz, H-8''''), 5.36 (1H, d, *J*=1.6 Hz, H-1''), 5.23 (1H, d, *J*=1.7 Hz, H-1'''), 5.15 (1H, t, *J*=9.5 Hz, H-4'), 4.45 (1H, d, *J*=7.3 Hz, H-1'), 4.12 (1H, m, Hb-8), 3.93 (1H, m, Ha-8), 3.11 (2H, t, *J*=6.7 Hz, H_2-7), 1.31 (3H, d, *J*=6.5 Hz, H_3-6''), 1.23 (3H, d, *J*=6.4 Hz, H_3-6''')

^{13}C-NMR (CD_3OD, 150 MHz) δ: 168.7 (C=O), 156.9 (C-4), 150.7 (C-4′′′′), 148.3 (C-7′′′′), 146.9 (C-3′′′′), 131.6 (C-2, 6), 130.8 (C-1), 127.7 (C-1′′′′), 124.5 (C-6′′′′), 117.3 (C-5′′′′), 116.5 (C-3, 5), 115.7 (C-2′′′′), 115.1 (C-8′′′′), 105.1 (C-1′), 104.3 (C-1″), 102.5 (C-1‴), 81.9 (C-4″), 81.4 (C-3′), 76.9 (C-5′), 76.2 (C-2′), 75.1 (C-4‴), 74.2 (C-2‴), 72.9 (C-3‴), 72.5 (C-2″), 72.2 (C-8), 71.1 (C-3″), 70.2 (C-4′), 69.4 (C-5″, 5‴), 63.1 (C-6′), 37.3 (C-7), 19.2 (C-6″), 18.3 (C-6‴)

Compound 3：Ligupurpuroside J (LPJ)

ESI-MS m/z: 759 [M-H]$^-$, Molecular formula: $C_{35}H_{46}O_{19}$

^{1}H-NMR (CD_3OD, 600 MHz) δ: 7.58 (1H, d, *J*=15.9 Hz, H-7""), 7.05 (1H, d, *J*=1.9 Hz, H-2""), 6.89 (1H, dd, *J*=8.4, 1.9 Hz, H-6""), 6.78 (1H, d, *J*=8.4 Hz, H-5""), 6.69 (1H, d, *J*=2.1 Hz, H-2), 6.64 (1H, d, *J*=8.2 Hz, H-5), 6.56 (1H, dd, *J*=8.0, 2.0 Hz, H-6), 6.28 (1H, d, *J*=15.8 Hz, H-8""), 5.19 (1H, s, H-1"), 5.18 (1H, s, H-1'''), 4.51 (1H, dd, *J*=12.0, 1.8 Hz, H-6'a), 4.35 (1H, d, *J*=8.1 Hz, H-1'), 4.34 (1H, *J*=11.9, 6.3 Hz, H-6'b), 4.13 (1H, dd, *J*=9.5, 6.2 Hz, H-5"), 3.98 (1H, m, H-2"), 3.98 (1H, m, H-8a), 3.87 (1H, m, H-2'''), 3.87 (1H, m, H-3"), 3.74 (1H, m, H-8b), 3.73 (1H, m, H-5'''), 3.58 (1H, m, H-3'''), 3.56 (1H, m, H-5'), 3.54 (1H, m, H-4"), 3.51 (1H, m, H-3'), 3.43 (1H, m, *J*=9.9 Hz, H-4'), 3.42 (1H, m, H-4'''), 3.33 (1H, m, H-2'), 2.78 (1H, t, *J*=7.5 Hz, H-7), 1.28 (3H, d, *J*=6.5 Hz, H-6'''), 1.26 (3H, d, *J*=6.5 Hz, H-6")

^{13}C-NMR (CD_3OD, 150 MHz) δ: 169.7 (C-9""), 150.2 (C-4""), 147.8 (C-7""), 146.9 (C-3""), 146.3 (C-4), 144.9 (C-3), 132.1 (C-1), 127.6 (C-1""), 124.3 (C-6""), 121.5 (C-6), 117.8 (C-2), 116.8 (C-5), 116.4 (C-5""), 115.7 (C-2""), 115.1 (C-8""), 105.1 (C-1'), 103.5 (C-1"), 102.8 (C-1'''), 83.4 (C-3'), 81.9 (C-4"), 75.9 (C-2'), 75.3 (C-5'), 74.1 (C-4'''), 73.5 (C-2'''), 73.1 (C-3"), 72.9 (C-8), 72.6 (C-2"), 72.3 (C-3'''), 71.1 (C-4'), 70.2 (C-5'''), 68.5 (C-5"), 65.1 (C-6'), 37.2 (C-7), 19.3 (C-6"), 17.5 (C-6''')
