# Supplementary material for: Wheat Starch Modified with Ligustrum robustum (Rxob.) Blume Extract and Its Action Mechanism
Source: Foods. 2022 Oct 13;11(20):3187. doi: 10.3390/foods11203187 (PMC9601434; doi:10.3390/foods11203187)
Supplement: Supplementary file 1 [file foods-11-03187-s001.zip › Supplementary File S2.pdf]

The detailed methods

1. Preparation of wheat starch and L. robustum (Rxob.) Blume extract

For the preparation of wheat starch, wheat flour was mixed with enough water to form a dough, then the dough was washed many times by water until faint yellow gluten was completely removed. Thereafter, the washed water was collected and centrifuged at 4000 r/min for 10 min at 25 °C, and then the precipitate was dried at 45 °C for 12 h to obtain the wheat starch

For the preparation of *L. robustum* (Rxob.) *Blume* extract, the dried sample of *L. robustum* (Rxob.) Blume was slightly ground (JYL-350, Jiuyang Co., Ltd., Jinan, China) and the sample powder (750 g) was added into water (5500 mL) with continuously stirring at 25 °C for 24 h. Then, the mixture was filtered and the filtrate was vacuum condensed to around 2000 mL at 45 °C by using a rotary evaporator (RE-3000A, Yarong, Co. Ltd., Shanghai, China). Thereafter, LRE was prepared by freeze-drying at -65 to -55 °C for 48 h, and then stored at 4 °C.

2. Determination of thermodynamic property

The thermal property of wheat starch was measured according to the previous study by using a Mettler-Toledo differential scanning calorimeter (DSC, Pyris/Diamond, Mettler Toledo International Trading (Shanghai) Co., Ltd, Shanghai, China). Briefly, 4 μL of LRE solution (0%, 2.5%, 5% and 10%, *w*/*v*) was mixed with 2 mg of wheat starch in the aluminum crucible, respectively. Subsequently, the aluminum crucible was sealed and incubated at 20 °C for 24 h, and then heated in the DSC equipment from 20 °C to 100 °C with a rate of 10 °C/min. Afterward, the gelatinization onset

temperature (T_o), peak temperature (T_p), conclusion temperature (T_c) and gelatinization enthalpy (ΔH_g) were recorded, and an empty aluminum crucible was used as control.

3. Determination of gelatinization property

The gelatinization property of wheat starch was evaluated by a Rapid Visco Analyzer (RVA, Perten Instruments of Australia Pty Ltd., Warriewood, Australia). Briefly, LRE was mixed with wheat starch (3 g) to reach the final concrntration of 0%, 5%, 10% and 20% (*w*/*w*, based on the weight of WS), respectively. Then, the mixture was evenly dispersed in 25 mL of water in RVA canister. Thereafter, all testing samples were equilibrated at 50 °C for 1 min, heated to 95 °C in 3.7 min, held at 95 °C for 2.5 min, then cooled to 50 °C in 3.8 min, and held at 50 °C for 2 min. The rotational speed was set at 960 r/min for the first 10 s, and then changed to 160 r/min. The gelatinization parameters of wheat starch, including peak viscosity (PV), hold viscosity (HV), break down (BD = PV−HV), final viscosity (FV) and pasting time (PT), were observed and recorded.

4. Determination of rheology property

The rheological property of wheat starch was determined by a stress controlled rheometer (AR G2; TA Instruments, New Castle, DE, USA) equipped with a parallel-plate geometry (40 mm in diameter, 1 mm in gap). Briefly, the wheat starch paste taken from the RVA experiment was loaded and kept on the plate for 5 min to achieve thermal balance. The strain sweep tests were run at 1 Hz to determine the linear viscoelastic range (LVR) of the sample. Thereafter, a strain of 1% within the

LVR was adopted to preform dynamic frequency sweep procedure from 0.1 to 100 rad/s , and the temperature of rheological experiment was set at 25 °C. Then, the rheological parameters of wheat starch, including the storage modulus (G′), the loss modulus (G″) and the loss tangent (Tanδ = G″/G′) against angular frequency (rad/s). were obtained.

5. Observation of gel microstructure

Briefly, LRE was added into wheat starch slurry (12%, *w/v*) to reach the final concentration of 5%, 10% and 20% (*w/w*, based on the weight of starch in solution), respectively. Thereafter, the mixture was boiled in a water bath for 30 min with continuous stirring and then rapidly cooled to 25 °C. Then, the mixture was stored at 4 °C for 1 d to obtain cylindrical gel (25 mm in height, 40 mm in diameter). After that, the starch gel samples were freeze-dried at -65 to -55 °C for 48 h, and all lyophilized samples were cut into pieces (1 cm × 1 cm × 0.5 cm). The surface of the testing sample was exposed to gold sputtering, and the microstructure was observed by the scanning electron microscope (SEM, SU8010, Hitachi, Ltd., Tokyo, Japan) at an accelerating voltage of 15 kV within a magnification of 250 times or 500 times.

6. Determination of wide-angle X-ray diffraction

The wide-angle X-ray diffraction of lyophilized samples prepared in section 5 was performed according to the previous study with some modifications by using a wide-angle X-ray diffractometer (D8 Advance, Bruker, Ltd., German). Prior to the test, the freeze-dried sample was pulverized to pass through a 60-mesh sieve, and native wheat starch was used as control. The instrument was operated at 40 kV and 40 mA

with CuKα radiation (λ = 0.154 nm). The scanning was applied at a diffraction angle (2θ) from 5° to 32° at a rate of 4°/min and the step size was set at 0.02°. MDI Jade 6.0 (version 6.0 for Windows, Materials Date, Inc., USA) was used to analyse the diffractograms. And the relative crystallinity was calculated as [crystalline area/(crystalline area + amorphous area)] × 100%.

7. Determination of color of biscuit

The color of biscuit was determined using a colorimeter (Konica Minolta, Chroma Meter, CR400, Japan). The instrument was standardized with standard white plates, and the $L*$, $a*$, and $b*$ values of biscuit samples were measured and recorded. An average value was calculated by taking observations from five samples in the same experimental group.

8. Determination of texture of biscuit

The texture of biscuit sample was determined according to the previous study with some modifications by using a TA-XT express texture analyzer (TA-XT2, Stable Micro Systems Ltd., Haslemere, UK) with a P/2N probe. Briefly, the pre-test speed, test speed, and post-test speed were set at 1.0, 0.5, and 10.0 mm/s, respectively. Meanwhile, the penetration distance was 5.0 mm, trigger force was 25.0 *g*. The hardness and fracturability values for all samples were determined. Five replicates for each experimental group were carried out.

9. Molecular dynamics simulation

Molecular dynamics (MD) simulation was used to analyse the interaction between LRE and wheat starch. For all the simulations, the starch model consisted of two

parallel short-chain glucose (SGS, each SGS contains 3 left-handed helixes formed with 18 D-glucopyranose linked by α-1,4-glycosidic bonds) and built by using the web of GLYCAM (https://www.glycam.org), the phenolic components identified from LRE (including LGB, LGN and LPJ) and reaction solvent (water solvent box, 65 × 46 × 49 $Å^3$) were established and optimized by the software of ChemBio3D Ultra (version 12.0 for Windows, PerkinElmer Inc., Waltham, Massachusetts, USA), Discovery Studio (version 4.5 for Windows, BIOVIA Inc., San Diego, CA, USA) and TIP3PBOX type of AMBER (version 18.0 for Linux, University of California., San Francisco, CA, USA), respectively. Thereafter, double chains of SGS with the GLYCAM-06j-1 force field and LGB (LGN or LPJ) with the generalized amber force field (GAFF) were loaded into the water solvent box in MD simulation system of AMBER, respectively. Afterward, the energy minimization procedure was separately performed to minimize the energy of water solvent box and the whole reaction system, which could optimize the reaction system energy.

For simulating the interactions between SGS and LGB (LGN or LPJ) during gelatinization and retrogradation, the NVT (canonical ensemble) procedure was carried out to increase (from 0 to 370 K) or decrease (from 370 to 277 K) the simulated temperature for 5×10^{-11} s, respectively. When NVT procedure ended, the NPT (constant molecule, pressure, and temperature) procedure was run to balance the simulated system in 5×10^{-11} s. With the stable reaction conditions, interactions between SGS and LGB (LGN or LPJ) in simulated gelatinization temperature (370 K) or simulated retrogradation temperature (277 K), such as the amount and changes of

binding site, type and strength of binding force, and the stability of binding action, were separately observed, recorded and calculated in 1×10^{-8} s (containing 5000 frames of trajectory). Meanwhile, the action of hydrogen atom was limited by adopting SHAKE rule and the step time was set to 2×10^{-15} s. The visual molecular dynamics (VMD) software (version 1.9.3 for Windows, Beckman Institute., Urbana, IL, USA) was used to obtain all visual schematic snapshots, and CPPTRAJ program was carried out for trajectory processing. All MD simulation procedures were carried out with AMBER software.
